# Photovoltaic Microorganism Hybrid Systems for Enhanced Polyhydroxybutyrate Synthesis Through Material Design and Energy Mass Transfer Mechanisms

**DOI:** 10.3390/ma19010001

**Published:** 2025-12-19

**Authors:** Jingyi Teng, Xinyi Chen, Hanyu Gao, Kaixin Huangfu, Silin Wu, Zhuo Ma, Ruiwen Wang, Shaoqin Liu, Yunfeng Qiu

**Affiliations:** 1 Faculty of Life Science and Medicine, School of Medicine and Health, Harbin Institute of Technology, Harbin 150080, China; 2024111349@stu.hit.edu.cn (J.T.); 2024111307@stu.hit.edu.cn (H.G.); 25b928069@stu.hit.edu.cn (K.H.); 25b928070@stu.hit.edu.cn (S.W.); shaoqinliu@hit.edu.cn (S.L.); 2 Key Laboratory of Bio-Based Material Science & Technology, Ministry of Education, Material Science Engineering College, Northeast Forestry University, Harbin 150001, China; c15082691457@163.com (X.C.); wrwnefu@163.com (R.W.); 3 Faculty of Life Science and Medicine, School of Life Science and Technology, Harbin Institute of Technology, Harbin 150001, China

**Keywords:** polyhydroxybutyrate, photosensitive nanomaterials, energy and mass transfer mechanisms, NADPH regeneration, light-driven biomanufacturing technologies

## Abstract

Polyhydroxybutyrate (PHB), as a biodegradable and green polymer, holds significant potential for replacing traditional petroleum-based plastics. However, its production efficiency and cost remain bottlenecks limiting large-scale application. In recent years, hybrid systems constructed from photosensitive nanomaterials and microorganisms have provided a novel pathway for enhancing PHB synthesis efficiency. These systems augment the supply of intracellular reducing power through efficient photo-generated electron injection, thereby driving microbial carbon fixation and PHB anabolic metabolism. This review systematically summarizes the mechanisms and performance of various types of photosensitive materials (including g-C_3_N_4_, CdS, polymer dots, etc.) in regulating PHB synthesis in microorganisms, such as *Cupriavidus necator* H16. It focuses on the influence of material composition, structure, energy band characteristics, and their interfacial interactions with microorganisms on electron transfer efficiency and biocompatibility. Furthermore, the article outlines the current challenges faced by these hybrid systems in key energy and mass transfer processes, including light energy conversion, transmembrane electron transport, and NADPH regeneration. It also prospects the design principles of novel bio-inspired multi-level heterojunction materials and their application potential in constructing efficient “material microbe” collaborative synthesis systems. This review aims to provide a material-level theoretical foundation and design strategies for developing high-performance and sustainable light-driven biomanufacturing technologies for PHB.

## 1. Introduction

Globally, advancing the circular economy and bio-manufacturing has emerged as a critical strategy for fostering green recovery, addressing climate change, and enhancing resource security. The 14th Five-Year Plan period has seen China explicitly accelerate the development of its circular economy and bio-manufacturing capabilities, aiming to enhance resource efficiency and achieve a green, low-carbon transition. Building on this momentum, the forthcoming 15th Five-Year Plan is poised to further emphasize these strategic areas, ensuring they remain central to the country’s sustainable development agenda [1]. Among the array of biodegradable plastics, such as polylactic acid (PLA), poly(butylene adipate-co-terephthalate) (PBAT), and poly(butylene succinate) (PBS), microbial-derived polyhydroxybutyrate (PHB) stands out due to its exceptional biodegradability and biocompatibility. Unlike PLA, which requires specific industrial composting conditions to degrade, PHB can be completely mineralized in a wider range of environments, including soil and marine settings, offering a definitive solution to plastic pollution [2]. However, despite this environmental promise, the industrial-scale production of PHB is hampered by challenges such as high production costs, limited product yield, and low substrate conversion efficiency, which currently restrict its market competitiveness and widespread adoption [3].

In recent years, with the increasing integration of nanomaterials and synthetic biology, light-driven hybrid systems based on the synergy of “semiconductor materials–microorganisms” have emerged, offering novel strategies to overcome the metabolic bottlenecks in PHB biosynthesis. These systems utilize photogenerated electrons from photosensitive nanomaterials under illumination to directly or indirectly enhance the intracellular reducing power (e.g., NADPH levels) of microorganisms, thereby activating or augmenting their carbon fixation and polymer synthesis capabilities. Studies have demonstrated that materials such as graphitic carbon nitride (g-C_3_N_4_), cadmium sulfide (CdS), and organic polymer dots (Pdots) can function as exogenous photosensitizers when integrated with PHB-producing strains (e.g., *Cupriavidus necator* H16, denoted as *C. necator* H16), establishing novel platforms for converting light energy into chemical energy to enable efficient PHB synthesis from CO_2_ or organic carbon sources [4]. Employing these photosensitive materials, the PHB production was enhanced to 6.73 g/L, representing an approximate 1.4- to 2.12-fold increase over the original yield [5].

Nevertheless, this field still faces multiple challenges, including limited material photochemical activity, insufficient biocompatibility, unclear electron transfer pathways, and poor system operational stability. In particular, traditional photosensitive materials commonly suffer from issues such as excessively large size, difficulty in traversing cell membrane barriers, and low electron utilization efficiency, which severely restrict their application potential in practical biomanufacturing processes. Therefore, systematically elucidating the mechanisms of photosensitive materials in microbial PHB synthesis and the principles for their design, summarizing current research progress on material microbe interface regulation and energy/mass transfer, and outlining the development directions for novel bioinspired, multidimensional heterostructure photosensitive materials hold significant scientific and engineering importance for advancing the practical application and industrialization of next-generation photo-driven biomanufacturing technologies [6].

The work by the Wang team has demonstrated the effectiveness of employing photoelectrocatalytic composite material systems in optimizing microbial metabolic pathways and enhancing electron transfer efficiency [7]. However, we observed that current reviews related to the bioplastic PHB predominantly focus on its modification and applications [8], PHA blending to enhance physicochemical properties, improving PHB yield through various metabolic engineering strategies in microorganisms [9], and optimizing fermentation conditions for higher productivity [10]. A relatively comprehensive review specifically addressing the integration with inorganic materials is notably absent. Therefore, to address this gap, this review aims to systematically elucidate the fundamental principles governing energy and mass transfer at the material microbe interface and to establish a coherent framework for understanding their role in driving PHB biosynthesis. Through an in-depth analysis of the processes encompassing electron injection, transmembrane transport, and metabolic coupling, we seek to distill novel design principles for next-generation photosensitive materials. Furthermore, we prospect the potential of advanced material systems, including bioinspired, multidimensional, and heterojunction architectures, for overcoming current bottlenecks. This work intends to provide a unified theoretical foundation for addressing the core challenge of low interfacial electron transfer efficiency, thereby laying a solid scientific and engineering basis for constructing high-performance artificial photosynthetic systems for PHB production.

## 2. Methodology

This review is structured to provide a systematic and critical analysis of the emerging field of photovoltaic microorganism hybrid systems for PHB production. The methodology underpinning this review is defined by its purpose, scope, function, and analytical intent. The primary purpose is to synthesize and evaluate the current understanding of how photosensitive nanomaterials interface with microbial metabolism to enhance the synthesis of PHB, with a particular focus on elucidating the energy mass transfer mechanisms that govern system efficiency. This involves consolidating fragmented knowledge from interdisciplinary studies spanning materials science, microbiology, and bioengineering to establish a coherent theoretical framework.

The scope of this review is deliberately focused on hybrid systems that integrate semiconducting materials (both inorganic and organic) with specific PHB-producing microorganisms, most notably *C. necator* H16. It encompasses a critical examination of material design principles, electron transfer pathways at the material microbe interface, the central role of NADPH regeneration, and the subsequent metabolic coupling that drives carbon fixation and polymer synthesis. The literature covered was selected to highlight the evolution from fundamental material concepts to advanced, bio-inspired heterostructures, providing a clear trajectory of technological progress. The function of this analysis is threefold: it is descriptive in summarizing key experimental findings and material performances; comparative in contrasting the advantages and limitations of different classes of photosensitizers; and prospective in identifying prevailing research gaps and forecasting future directions. Finally, the analytical intent is mechanism-oriented, seeking to establish clear structure property function relationships that link material characteristics (e.g., size, band gap, surface chemistry) to biological outcomes (e.g., NADPH/NADP^+^ ratio, PHB yield) [11]. This critical approach aims not only to inform the scientific community but also to distill practical design strategies for developing the next generation of high-performance, light-driven biomanufacturing systems.

## 3. Overview of Poly-β-hydroxybutyrate

PHB is a biopolymer synthesized by bacteria under specific conditions, such as nitrogen limitation [12]. As a member of the polyhydroxyalkanoates (PHA) family, PHB is a biodegradable material exhibiting thermoplastic properties. It possesses a high melting point of approximately 177–180 °C, a glass transition temperature of about 2 °C, and a molecular weight typically ranging from 10^6^ to 10^7^ Da. PHB demonstrates a tensile strength of 43 MPa and a crystallinity exceeding 60% [13]. It is insoluble in water, ethanol, and acetone but soluble in chlorinated organic solvents like chloroform and dichloroacetic acid [14]. Owing to its biodegradability and environmentally benign characteristics, PHB has garnered significant attention as a potential substitute for conventional petroleum-based plastics. Physiologically, PHB serves as a primary carbon storage material for bacteria; it can be degraded into acetyl-CoA for energy metabolism under nutrient starvation and also functions in regulating cellular osmotic pressure. In the materials sector, PHB is extensively utilized in applications such as packaging materials, drug delivery carriers, and agricultural films due to its biodegradability and biocompatibility.

**Figure 1:**
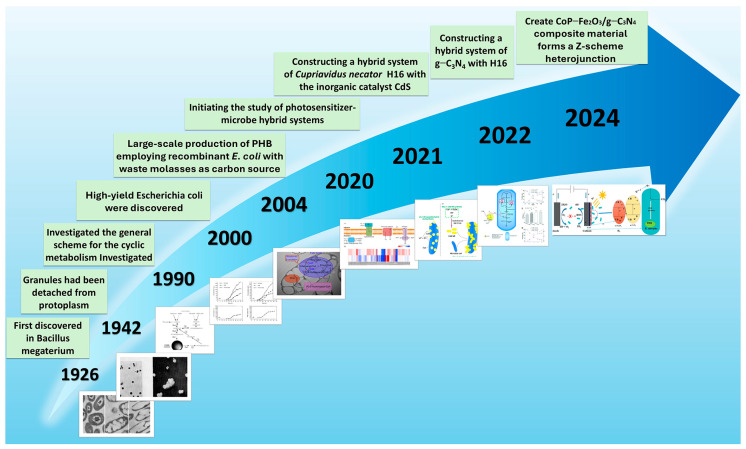
Timeline of the development of PHB biosynthesis.

Figure 1 outlines the key milestones in PHB research, tracing its evolution from the initial discovery in Bacillus megaterium in 1926, through large-scale production using recombinant *E. coli* with low-cost feedstocks such as molasses, to the recent emergence of photosensitizer-microbe hybrid systems. This progression reflects a significant paradigm shift from traditional microbial fermentation toward material-driven, light-enhanced biosynthetic strategies. The evolutionary pathway encompasses foundational studies on inorganic catalysts, exemplified by cadmium sulfide coupled with *C. necator* H16, followed by the introduction of organic semiconductors such as graphitic carbon nitride (g-C_3_N_4_), and ultimately advances toward more efficient PHB production through composite photosensitive materials like CoP-Fe_2_O_3_/g-C_3_N_4_ integrated with *C. necator* H16.

**Figure 2:**
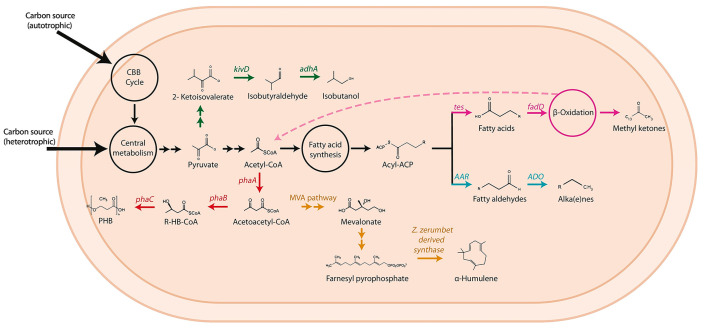
Redirecting carbon flux from PHB synthesis in *C. necator* H16 [29]. Copyright 2021 Elsevier Ltd. All rights reserved.

Several methods exist for PHB synthesis, including microbial fermentation (Table 1), transgenic plant approaches, chemical synthesis, and chemo-enzymatic hybrid methods [15]. Among these, microbial fermentation is undoubtedly the most promising, as it does not require cumbersome and complex genetic modifications, hazardous and highly polluting chemical synthesis, or expensive enzymes as reaction media [16]. However, bacterial fermentation remains expensive due to fermentation substrates and the complex fermentation process, making it unable to compete with chemically synthesized plastics [17]. Therefore, finding ways to reduce the cost of fermentation substrates, identifying more economical substrate sources, or simplifying the fermentation process have become pressing issues that need urgent resolution. The bacterium *C. necator* H16 has garnered widespread attention as a natural strain capable of producing PHB using CO_2_, particularly under nitrogen-limiting conditions [18]. It can autotrophically produce PHB with CO_2_ as the carbon source, or heterotrophically produce it using carbon sources such as fructose. Although *C. necator* H16 possesses the inherent ability to utilize CO_2_ for PHB production, its production efficiency, especially under ambient pressure conditions, is generally low [19]. Studies on its PHB metabolic pathway reveal that *C. necator* H16 primarily fixes CO_2_ through the Calvin-Benson-Bassham (CBB) cycle (Figure 2). The fixed carbon subsequently enters the central carbon metabolism, where it is converted into pyruvate by relevant enzymes.

This process demands substantial amounts of NADPH and ATP to supply reducing power and energy [30]. Concurrently, within the cell, pyruvate is first converted to acetyl-CoA. Two molecules of acetyl-CoA are then condensed by 3-ketothiolase (PhaA) to form acetoacetyl-CoA. Subsequently, acetoacetyl-CoA is reduced by acetoacetyl-CoA reductase (PhaB) to (R)-3-hydroxybutyryl-CoA. Finally, PHA synthase (PhaEC) polymerizes (R)-3-hydroxybutyryl-CoA to form PHB. The reduction of acetoacetyl-CoA to (R)-3-hydroxybutyryl-CoA by PhaB also critically requires NADPH as a reducing equivalent. This specific step serves as the rate-limiting stage of the entire pathway, playing a pivotal role in determining the final PHB yield [31]. In other words, enhancing the availability of NADPH during this phase can effectively increase the overall production of PHB.

### 3.1. PHB Synthesis Methods and Application Fields

#### 3.1.1. Synthesis Methods of PHB

The main synthetic routes for PHB include chemical synthesis, microbial fermentation, transgenic plant methods, and chemo-enzymatic approaches (Table 2). Chemical synthesis typically employs β-butyrolactone as feedstock, building PHB chains through ring-opening polymerization. This method enables precise control over polymer structure, molecular weight, and terminal functional groups, facilitating customized material design. However, its reliance on petrochemical derivatives and metal catalysts imposes limitations on sustainability and environmental compatibility. Transgenic plant strategies aim to incorporate PHB biosynthesis into plant systems, potentially utilizing photosynthesis for direct carbon fixation into polymers. Despite cost reduction potential, this approach confronts multiple challenges: low polymer yield, adverse effects on plant growth, transgene instability, and high extraction costs, hindering industrial implementation. The chemo-enzymatic hybrid method represents an emerging approach, exemplified by chemical hydrogenation of CO_2_ to methanol followed by enzymatic conversion to PHB. While demonstrating advantages in atom economy and carbon emission reduction, technical barriers persist in multi-enzyme system coordination, catalyst cost and stability, and process integration. Microbial fermentation utilizes microbial cells as natural bioreactors, converting renewable carbon sources such as sugars, lipids, and even industrial waste into PHB through their inherent metabolic networks. Its prominent advantages include the use of inexpensive substrates for scalable production and products with excellent biocompatibility and complete biodegradability. Compared to other methods, microbial fermentation operates under mild conditions without requiring high temperature, high pressure, or toxic catalysts, aligning with green chemistry principles. However, it faces challenges such as high production costs and relatively low yields. Consequently, current research is exploring the use of modern synthetic biology and metabolic engineering tools to modify microbial strains. These approaches enable the regulation of polymer molecular weight, the synthesis of novel copolymers, and the combination of microbes with photosensitive materials to enhance electron transfer efficiency, increase PHB productivity, and reduce production costs.

#### 3.1.2. Comparison of PHB with Other Bioplastics

Among the prominent bioplastics in commercial use today, including PLA, PBAT, PBS, and PHB, PLA is the most widely adopted, representing approximately 32% of global bioplastic production [35]. Its market dominance, however, is attributable not to superior environmental performance but to more mature industrial scalability, processing characteristics, and a well-established supply chain. In contrast, PHB has seen limited commercial penetration, mainly due to its high production costs and inherent brittleness (Table 3). A significant drawback of PLA is its restricted biodegradability. Although it degrades efficiently under controlled industrial composting conditions within about 180 days, its breakdown in natural environments such as soil or marine ecosystems is considerably slow, leading to persistence comparable to conventional petroleum-based plastics [36]. This limitation reduces its effectiveness in mitigating plastic pollution in unmanaged environments. PHB, as a principal member of the PHA family, offers a distinct advantage in its broad biodegradability. It can be fully mineralized by microorganisms across diverse environments including soil, freshwater, and marine habitats [37]. For example, studies on poly(3-hydroxybutyrate-co-3-hydroxyvalerate) (PHBV), a common PHB copolymer, have shown extensive microbial colonization and degradation in marine settings [38]. This capacity for complete environmental assimilation makes PHB a more robust material for reducing long-term plastic accumulation. From a production perspective, although both polymers are bio-derived, their synthesis pathways differ considerably in complexity and sustainability potential. PLA is typically produced via fermentation of plant-based sugars into lactic acid, followed by chemical polymerization [38]. This process often requires sterile conditions and purified feedstocks, increasing energy and cost inputs [39]. In contrast, PHB can be synthesized by mixed microbial cultures using organic waste streams, such as food waste, as carbon sources [40]. This approach not only lowers production costs but also supports circular economy principles by upcycling waste materials [41]. In summary, while PLA’s current market leadership is driven by economic and processing advantages, PHB possesses stronger long-term potential due to its proven, comprehensive biodegradability and potential for sustainable production from waste resources (Table 4). Future development focusing on copolymerization or biocomposites may effectively address PHB’s current limitations in cost and mechanical properties, positioning it as a highly promising material for advanced bioplastic applications.

Therefore, reducing the production cost of PHB bioplastics and enhancing their production efficiency to meet market demand for environmentally friendly materials have become key industry drivers for this research [42]. Concurrently, the utilization of waste pollutants and CO_2_ as feedstocks for bioplastic production has become feasible. These breakthroughs enable the establishment of systems that utilize CO_2_ or other biomass waste as carbon sources for fermentative bioplastic production. The development of such systems not only addresses the environmental pollution issues associated with the disposal of CO_2_ and other biomass waste but also produces bioplastics that are versatile, environmentally benign, and recyclable [43]. This holds significant importance for achieving the dual carbon strategy and promoting the efficient development of the biodegradable bioplastics industry [44].

#### 3.1.3. Modification Strategies and Application Fields of PHB

PHB is regarded as an ideal substitute for conventional petroleum-based plastics due to its biodegradability and biocompatibility, showing broad application prospects in fields such as biomedical, packaging, and agriculture [45]. However, the inherent brittleness of pure PHB, characterized by low tensile yield strength (typically below 10 MPa) and minimal elongation at break (approximately 5%), along with insufficient impact strength, severely limits its commercial application and widespread adoption [46]. To address these limitations, researchers have developed various modification strategies aimed at enhancing the properties of PHB to meet broader application requirements [47]. Currently, four primary modification approaches are employed: blending modification [48], chemical modification [49], additive modification, and in vivo modification strategies during biosynthesis.

Blending modification involves combining PHB with other polymers or materials—such as polylactic acid (PLA), microcrystalline cellulose (MCC), lignin, polyethylene glycol (PEG), cellulose nanocrystals (CNCs), starch [50], polycaprolactone (PCL) [4], zirconium dioxide (ZrO_2_), or amorphous medium-chain-length PHA (aPHA)—to effectively improve its mechanical strength, toughness, thermal stability, antibacterial properties, and processability. Chemical modification enhances PHB performance by introducing new chemical groups or altering the polymer structure [51]. For instance, photografting with PEG can increase the hydrophilicity of PHB, while grafting with maleic anhydride (MA) improves its mechanical, thermal, and rheological properties [52]. Additionally, post-polymerization modifications such as thiol-ene and phosphine addition reactions can be applied to PHB to impart new functionalities [53].

Additive modification utilizes various fillers and plasticizers to tailor the properties of PHB. For example, incorporating lauric acid (LA) can enhance the mechanical and thermal performance of PHB/starch composite films. The addition of fillers like carbon nanoparticles [54], functionalized few-layer graphene (FFG), and calcium sulfate whiskers (CSW) also improves the mechanical and thermal characteristics of PHB. In vivo modification during biosynthesis involves optimizing microbial fermentation conditions to control the molecular structure and composition of PHB, thereby enhancing its properties directly at the polymerization stage.

Through these modifications, PHB materials leverage their inherent biocompatibility and biodegradability, coupled with enhanced mechanical properties, to meet the demands of a wider range of applications.

##### Biomedical and Tissue Engineering Applications

PHB and its modified derivatives hold significant application value in the biomedical field. PHB surfaces modified via photografting with PEG exhibit significantly enhanced hydrophilicity, accelerating their biodegradation rate in enzymatic environments while preserving their bulk mechanical properties [55]. This is crucial for medical implants requiring specific degradation profiles and good tissue compatibility.

Furthermore, two-dimensional substrates of PLA, PHB, or PHB-HV functionalized through aminolysis with multifunctional amines (such as 1,6-hexanediamine (HDA), tetraethylenepentamine (TEPA), and poly(allylamine hydrochloride) (PAH)) demonstrate excellent antifouling properties [56]. This antifouling capability is significant for medical devices and food packaging, helping to prevent biofilm formation and infections [57].

Incorporating ZrO_2_ into PHB enhances its antibacterial properties, which is particularly beneficial for developing medical implants resistant to biofilm formation. The addition of bioactive fillers like hydroxyapatite (HA) also indicates the potential of PHB composites in bone tissue engineering and related fields, improving both biocompatibility and mechanical performance.

##### Packaging Materials

The biodegradability of PHB makes it an ideal substitute for conventional plastic packaging [58]. However, the inherent brittleness of pure PHB limits its packaging applications. Blending PHB with other biodegradable polymers, such as PLA and PCL, can effectively enhance its mechanical properties and processability, rendering it suitable for food packaging and bioabsorbable medical applications. Ternary blends of PHB, PLA, and PCL allow for the modulation of degradation behavior and mechanical properties by adjusting their composition, demonstrating good biodegradability in marine environments and thereby helping to mitigate plastic pollution [59]. Furthermore, composite films incorporating starch as a low-cost biomass filler and LA as a plasticizer exhibit improved mechanical and thermal performance, further advancing the commercial viability of PHB in the packaging sector.

##### 3D Printing Applications

Modified PHB materials show significant potential in the field of 3D printing, particularly for fused deposition modeling (FDM) technology. PHB/PLA blends can be utilized in FDM 3D printing, and the recycling of PHB/PLA regenerated pellets helps reduce plastic waste and enables sustainable production [60]. Composites prepared by blending PHB with poly(butylene adipate-co-terephthalate) (PBAT) and nanofibrillated cellulose (NFC) significantly enhance the thermal and mechanical properties of the material, making it more suitable for FDM 3D printing applications [61]. Furthermore, carbon nanoparticles extracted from waste coconut shells (CCSP10) can be used as fillers to modify BIOPLAST GF 106/02/PLA 75/25 blends, producing eco-friendly bioplastic polymer filaments that provide high-performance, sustainable materials for 3D printing.

##### Agricultural Applications

Modified PHB holds broad application prospects in agriculture. Its biodegradable characteristics make it suitable for agricultural mulch films and controlled-release fertilizer coatings. Modified PHB films can replace traditional plastic agricultural films, thereby reducing soil pollution. By adjusting the degradation rate of modified PHB, it can be aligned with crop growth cycles, enabling natural degradation after providing necessary functions and contributing to environmental protection [62].

##### Light Industry Sector Applications

The limited deformation capacity and narrow processing window of PHB restrict its widespread use. Incorporating linear-chain polyester oligomers as plasticizers can improve the processability and flexibility of PHB, while also reducing its glass transition temperature (T_g_), degree of crystallinity, crystallization rate, and melting point (T_m_) [63]. This facilitates the substitution of conventional plastics with PHB in a broader range of industrial products, such as disposable tableware, toys, and consumer product casings. Furthermore, blending PHB with 20% beech wood flour can produce fully biodegradable films. Pretreatment of the wood flour can enhance the interfacial adhesion within the composite, making it suitable for applications requiring specific structural strength [64].

### 3.2. Review of Current Research Status: Domestic and International

The large-scale production of PHB has long relied primarily on traditional microbial fermentation techniques. In recent years, with the increasing convergence of materials science and biomanufacturing, hybrid systems based on the synergy between photosynthetic materials and microorganisms have opened up a novel technological pathway for the efficient synthesis of PHB. This section will systematically review the developmental trajectory and current research status, from traditional bioprocessing to emerging photosynthetic material-microorganism hybrid systems [65].

#### 3.2.1. Development Overview of Traditional Bioprocessing Methods

Traditional bioprocessing represents the most established technological route for PHB production. Its development spans several decades and has achieved varying degrees of commercial application worldwide. As early as 1975, Imperial Chemical Industries (ICI) in the UK pioneered the use of an A. eutrophus mutant for PHB production, initiating the preliminary exploration of PHB industrialization. In 1981, Zeneca, a company under ICI, further utilized a glucose-utilizing mutant strain of A. eutrophus to produce PHB and its copolymer, PHBV, via batch fermentation. Research results indicated that under culture conditions with phosphorus limitation and other salts in excess, PHBV yield could reach 70% to 80% of the cell dry weight [66].

Subsequently, the Monsanto Company acquired the production patent rights for PHBV and successfully commercialized plastic products made from PHBV under the trade name “Biopol”, achieving an annual production capacity of 1000 tonnes [67]. The U.S.-based Berlin Packaging Company has begun supplying packaging bottles made from Biopol material to the hair care industry [68], signifying the initial market acceptance of PHB-based materials. In Europe, the Austrian company Chemie Linz GmbH developed a continuous fermentation process based on A. eutrophus, achieving a PHB production capacity of one tonne per week [69]. Other bio-chemical companies in the country subsequently achieved pilot-scale production of PHB. For instance, using Alcaligenes latus as the production strain and sucrose as the carbon source in a 15 m^3^ fermenter, the annual production capacity reached 20 tonnes [70]. Notably, researchers Ishizaki A. et al. utilized the autotrophic bacterium R. eutropha ATCC17697T for fermentation in an oxygen-limited fermenter using inorganic CO_2_ as the sole carbon source. After 60 h of cultivation, they achieved a high cell dry weight of 60 g/L and a PHB yield of 36 g/L [22], providing a significant demonstration for producing bioplastics using the greenhouse gas CO_2_. Research on PHA-based biodegradable plastics in China commenced relatively later. Institutions including Tsinghua University and the Institute of Microbiology, Chinese Academy of Sciences initiated related research in the early 1990s, successfully developing a PHB production process using waste molasses as the raw material at the laboratory scale [71]. Around 2004, Tsinghua University, led by Chen’s team, successfully developed the third-generation PHA—poly (3-hydroxybutyrate-co-3-hydroxyhexanoate) (PHBHHx), established the complete process chain from laboratory-scale, pilot-scale to industrial-scale production [72]. The team achieved remarkable results in producing PHB using recombinant *E. coli* and low-cost carbon sources like waste molasses. Several technologies developed by the team have been implemented for large-scale production by multiple companies [73], significantly advancing China’s bio-manufacturing industry. Furthermore, a team led by Wang, successfully demonstrated the conversion of TPA to PHA using shake-flask experiments to enrich mixed microbial communities from sludge, with terephthalic acid (TPA), a hydrolysis product of PET, serving as the carbon source. Their work also elucidated the dominant microbial community composition and the TPA conversion pathway [74].

#### 3.2.2. Limitations of Traditional Bioprocessing Methods

Despite significant advancements in traditional bioprocessing methods, their further development faces several bottlenecks [75]. Firstly, existing strategies primarily focus on genetic engineering modifications or strain screening of the microorganisms themselves, failing to introduce external driving forces to surpass their inherent metabolic limits [76]. Secondly, the fermentation process typically relies on expensive pure substrates or complex culture media, resulting in persistently high production costs. Most importantly, the regeneration efficiency of the intracellular reducing power NADPH often acts as the rate-limiting step for the PHB synthesis rate [49]. Consequently, developing novel technologies capable of directly intervening in and enhancing key intracellular metabolic processes has become crucial for surpassing the current yield ceiling.

#### 3.2.3. The Emergence of Novel Fermentation Strategies Using Photosynthetic Material Microbe Hybrid Systems

To overcome the limitations of traditional fermentation methods, researchers have turned their attention to an emerging interdisciplinary field focusing on photosynthetic material microbe hybrid systems [77]. The core of this strategy lies in combining inorganic or organic photosensitive materials with PHB-producing strains (e.g., *C. necator* H16) to directly enhance intracellular metabolic flux using photogenerated electrons. The fundamental scientific principle is that artificially improving the conversion efficiency from NADP^+^ to NADPH (i.e., accelerating the process by which NADP^+^ gains 2 e^−^ and H^+^ to be reduced to NADPH [78]) holds the potential to fundamentally alleviate the reductant limitation in the PHB synthesis pathway. By synergizing functionally photocatalytic materials with bacteria, the photogenerated electrons produced by the photosensitizers under illumination can be utilized to promote the efficient regeneration of NADPH, thereby increasing the operational rate of the entire PHB synthesis network and ultimately achieving a leap in production yield [79].

However, the traditional photosensitizers employed in the early stages of this research exhibited several significant drawbacks (Table 5). For instance, traditional carbon-based photosensitizers (e.g., g-C_3_N_4_) [80], while possessing good biocompatibility, generally suffer from low quantum yield of photogenerated electrons. In contrast, traditional non-carbon-based photosensitizers (e.g., CdS) exhibit high photocatalytic activity but poor biocompatibility [81]; the heavy metal ions they contain can severely inhibit microbial growth and activity during fermentation, potentially leading to decreased biomass and ultimately failing to enhance PHB production effectively. More critically, these traditional photosensitizers are typically large in size [82], and their interaction with microorganisms is confined to the outer cell surface. They must rely on slow and inefficient extracellular electron transfer processes to indirectly deliver electrons into the cell. This process often requires the additional, cumbersome step of adding exogenous electron mediators [83], making it complex and difficult to precisely regulate for efficiency [84].

These challenges render the design and construction of novel photocatalytic nanomaterials possessing excellent quantum yield of photogenerated electrons, superior biocompatibility, and ultrasmall size particularly urgent [85]. The ideal new material should have a sufficiently small size (typically <20 nm) to enable penetration of the cell membrane barrier and entry into the bacterial interior. In this way, photogenerated electrons can act directly on the reduction of NADP^+^ in situ within the cell, bypassing the need for complex transmembrane transfer processes [86]. This direct intracellular action would achieve higher electron utilization efficiency, significantly accelerate NADPH regeneration, and ultimately drive PHB synthesis with high efficiency [87].

Should this technological approach be successfully realized, it would have profound implications. Firstly, it could significantly enhance PHB production by *C. necator* H16 across various carbon sources. Whether utilizing pure CO_2_, industrial waste gases, fructose, glucose, or even waste biomass, the yield efficiency is expected to substantially surpass that of traditional methods [88]. Secondly, this material microbe collaborative paradigm possesses general applicability and can be extended to other industrial strains with production potential. By acting precisely on specific biosynthetic pathways, it could further boost the yield of target products beyond existing baselines [89]. This signifies a novel strategy for directly enhancing microbial metabolic capacity through non-genetically modified means. Ultimately, this production model, which introduces functional materials to collaborate with microbes for improved efficiency and product quality, fully aligns with the characteristics of fourth-generation bioprocessing technologies [90]. It not only holds promise for resolving the cost and efficiency challenges of PHB production but could also provide powerful momentum for the technological upgrading of the entire bio-manufacturing sector, demonstrating vast potential for industrialization.

**Table 5:** Photosensitizers Promote PHB Production by *C. necator* H16.

Classification	Representative Materials	Band Gap (eV)	Light Absorption Range (nm)	PHB Yield	Main Advantages	Main Limitations	Experimental Parameters	Literature Source
Inorganic Semiconductors	CdS	2.4	400–520	1.41 g/L	Broad spectrum absorption High electron yield	Heavy metal toxicity, large size	Light Intensity: 4200 lx (LED) Wavelength: λ > 420 nm Carbon Source: Fructose (20 g L^−1^) or CO_2_	[91]
Organic Semiconductors	g-C_3_N_4_	2.7	400–460	6.73 g/L	Good biocompatibility Cost-effective	Easy electron-hole recombination	Light Intensity: 4200 lx (LED) Wavelength: λ < 450 nm Carbon Source: Fructose (20 g L^−1^)	[5]
Organic Semiconductors	Pdots	Tunable (2.3–2.8)	450–550	0.021 g/L	Small size Surface functionalizable	Long-term photostability needs to be improved	Light Intensity: 2.5 mW cm^−2^ (Optimized, Simulated Sunlight) Wavelength: Broad-spectrum Visible Light (AM 1.5G Filter) Carbon Source: CO_2_ (supplied as NaHCO_3_ in the medium)	[2]
Composite Materials	CoP-Fe_2_O_3_/g-C_3_N_4_	2.1–2.5	400–600	0.142 g/L	Broaden light absorption Promote charge separation	Complex preparation process	Light Intensity: 16 klux (Iodine-tungsten lamp) Wavelength: Visible Light Carbon Source: CO_2_	[92]
Representative Materials	Ni@CNTs	-	-	0.477 g/L	High electrical conductivity Large specific surface area	Dependence on external potential	Light Intensity/Wavelength: N/A (electrocatalysis-driven) Incubation Time: 7d Carbon Source: CO_2_	[93]

Note: The hyphen “-” indicates that the data is not available.

### 3.3. Photosynthetic Material Microbe Hybrid System Fermentation Methods

In light-driven bio-hybrid systems, photosensitive materials serve as the core components for light energy capture and electron transfer, and their performance directly determines the system’s efficiency in converting light energy into chemical energy [94]. Based on the characteristics of various photosensitive materials coupled with *C. necator* H16, they can be systematically categorized into three primary classes: inorganic semiconductors, organic semiconductors, and emerging nano-photosensitizers [95]. These distinct classes of materials exhibit significant differences in their energy band structures, quantum yields of electrons, and biocompatibility.

#### 3.3.1. Inorganic Semiconductor Photosensitizers

Inorganic semiconductor photosensitizers, represented by metal compounds, primarily include materials such as CdS [96]. These materials typically possess well-defined crystal structures, excellent charge carrier mobility, and a broad spectral response range. CdS has a band gap of approximately 2.4 eV, corresponding to an absorption edge around 520 nm, enabling it to effectively utilize photon energy in the visible light region [97].

**Figure 3:**
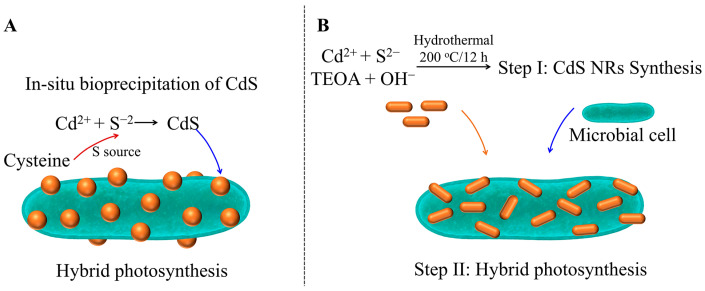
Schematic illustration of two strategies for constructing CdS-microbial hybrid photosynthesis systems. (**A**) In situ bioprecipitation: CdS nanoparticles are generated directly on the microbial cell surface using cysteine as the sulfur source. (**B**) Two-step assembly: CdS nanorods (NRs) are first synthesized via a hydrothermal method (200 °C, 12 h) and subsequently assembled with microbial cells to form the hybrid system. The green oval represents the microbial cell, and the orange spheres and rods represent the CdS nanoparticles and nanorods, respectively.

As illustrated in Figure 3, distinct strategies can be employed to construct CdS-microbial hybrids. Research by Xu et al. demonstrated that by adopting the two-step assembly strategy (Figure 3B) to optimize the morphology of CdS nanorods and coupling them with *C. necator* H16, the PHB yield increased from 0.68 g/L to 1.41 g/L under heterotrophic conditions with CO_2_ as the carbon source, the yield increased from 0.016 g/L to 0.028 g/L, a 1.74-fold increase [91]. This significant improvement is attributed to the excellent photogenerated electron production capability of CdS.

However, the practical application of such materials faces severe challenges: firstly, the leaching of Cd^2+^ ions exhibits significant toxicity to microorganisms, inhibiting cell growth and metabolic activity [98]; secondly, their relatively large particle size (typically >50 nm) hinders their ability to traverse the cell membrane, resulting in electron transfer being confined to the external cell surface and reliant on mediator molecules for indirect transfer, which limits efficiency and results in poor system [99].

The leaching of Cd^2+^ is the core mechanism underlying the toxicity of cadmium sulfide (CdS), and its mitigation can be achieved through various surface regulation and immobilization strategies: firstly, the physical barrier strategy, which directly blocks the contact channel between Cd^2+^ and the external environment by constructing coatings or passivation layers such as SiO_2_ and TiO_2_ [100]; secondly, the core-shell structure modification strategy, which utilizes materials including metal-organic frameworks (MOFs) and carbon quantum dots (CQDs) to form core-shell composite structures [101]. This strategy not only inhibits Cd^2+^ leaching through spatial shielding effect but also improves the comprehensive performance of the material via the functional synergy between the shell and core phases-for instance, in the CdS-bacteria hybrid system, the introduction of cystine as a sulfur source and surface capping agent enables efficient passivation of the CdS surface, significantly suppressing Cd^2+^ leakage induced by photocorrosion. Relevant studies have confirmed that the system can operate continuously and stably for several days, while the bacteria maintain intact metabolic activity and continuously synthesize acetic acid, fully verifying the effectiveness of surface chemical regulation in inhibiting CdS toxicity [100]; in addition, the chemical immobilization by chelating agents such as phytochelatin derivatives, or the immobilization technology using porous carriers like biochar, can further restrict the migration and diffusion of Cd^2+^ [102]. Multiple studies have demonstrated that the aforementioned strategies can effectively reduce the leaching concentration of Cd^2+^ (the leaching amount of some systems is reduced to 0.003-0.01 mg/L, meeting the drinking water quality standard), and their stability has been validated through long-term kinetic experiments (6–180 days); notably, some strategies such as core-shell modification can retain the core functions of CdS (e.g., photocatalysis) while inhibiting toxicity, and reduce the risk of chronic toxicity to microorganisms or plants. The application of these toxicity mitigation strategies provides important technical support for constructing CdS-microbe composite systems with higher stability and better environmental compatibility.

#### 3.3.2. Organic Semiconductor Photosensitizers

**Figure 4:**
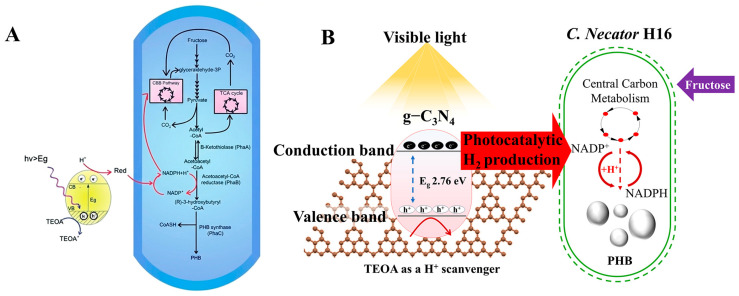
Hybrid photosynthesis optimization with g-C_3_N_4_ and *C. necator* H16 producing the bioplastic PHB from fructose and visible light. (**A**) Scheme for hybrid photosynthesis with g-C_3_N_4_ and fructose-fed *R. eutropha* [5], where red arrows represent the transfer of reducing equivalents. Copyright 2022 Trends in Biotechnology. All rights reserved. (**B**) Schematic mechanism of the hybrid system under visible light irradiation (yellow beam). g-C_3_N_4_ generates electron-hole pairs (*E_g_* = 2.76 eV), driving photocatalytic H_2_ production (large red arrow). This acts as an electron carrier to boost NADPH regeneration (red curved arrows) in *C. necator* H16. Combined with fructose metabolism (purple arrow), this additional reducing power significantly enhances the intracellular accumulation of PHB granules (white spheres). TEOA acts as the sacrificial hole scavenger.

Organic semiconductor photosensitizers, represented by g-C_3_N_4_ and Pdots, have garnered significant attention due to their favorable biocompatibility and tunable electronic structures.

g-C_3_N_4_ possesses a band gap of approximately 2.7 eV, corresponding to an absorption edge around 460 nm, enabling it to effectively utilize the blue-violet light region. The team of Xu first reported a hybrid system of g-C_3_N_4_ with *C. necator* H16, which increased the PHB yield from 4.9 g/L to 6.73 g/L under heterotrophic conditions with fructose as the carbon source over a 96-h fermentation period, representing an enhancement of approximately 1.4-fold [5] (Figure 4A). It is noteworthy that this material boosted production while exhibiting significantly less inhibition of cell activity compared to CdS. Wang et al. further investigated the influence of light intensity on the g-C_3_N_4_-*C. necator* H16 system (Figure 4B), finding that under 2100 lx illumination, the PHB production rate increased from 1.74 g/L/d to 3.7 g/L/d, a 2.12-fold improvement, with significant promotional effects observed across the range of 1200–6300 lx [103] indicates that g-C_3_N_4_ possesses good light-responsive characteristics and biocompatibility. However, g-C_3_N_4_ still suffers from issues such as a high charge carrier recombination rate and poor electrical conductivity, which limit its electron transfer efficiency.

**Figure 5:**
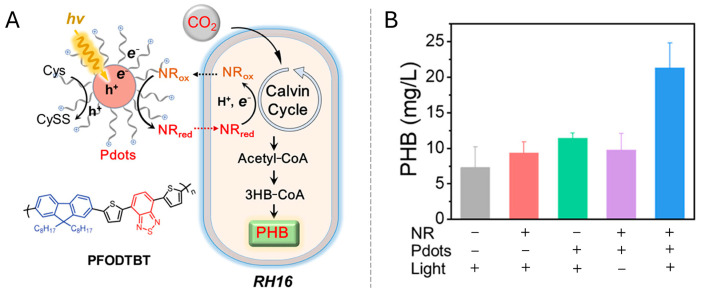
Performance and mechanism of the *RH16*/NR/Pdots photosynthetic biohybrid. (**A**) Proposed mechanism of NR-mediated electron transfer from Pdots to cellular metabolism [2]. (**B**) Quantification of PHB production under optimized conditions (20 μM NR, 0.02% Cys, 4 μg mL^−1^ Pdots, 2.5 mW cm^−2^, 12 h light/dark cycles, 30 °C, 2 days) [2]. Copyright 2022 ACS Publications. All rights reserved.

Pdots are promising organic nano-photosensitizers due to their large absorption cross-section and tunable energy levels. In a study by Yu, a biohybrid system incorporating Pdots and the electron shuttle neutral red (NR) with *Ralstonia eutropha* H16 achieved a PHB yield of 0.021 g/L from CO_2_, tripling the output of the control [1]. It is important to note that in this system, the Pdots (~70 nm) functioned extracellularly on the cell surface, with NR shuttling the electrons across the membrane (Figure 5). Despite their advantages, organic photosensitizers like Pdots often face challenges such as photobleaching and lower quantum yields compared to their inorganic counterparts.

Addressing the concern regarding the photostability of organic photosensitizers, particularly Pdots, is crucial for long-term PHB production. Integrating structurally constrained fluorophores, such as those based on the hexamethylazatriangulene motif, can significantly enhance the inherent photostability of these photocatalytic components by suppressing non-radiative decay and improving oxidative resistance [104]. Furthermore, photodegradation driven by photoinduced radicals, a known failure mechanism for Pdots, can be effectively mitigated through medium engineering [105]. Employing biocompatible buffers like HEPES or MES, which act as radical scavengers in the bacterial culture medium, has been shown to improve the photostability of semiconducting polymers significantly (e.g., >10-fold) [106]. This offers a simple yet effective strategy to protect Pdots within the hybrid system without compromising bacterial viability.

#### 3.3.3. Application of Composite Photoelectric Materials in Hybrid Systems

**Figure 6:**
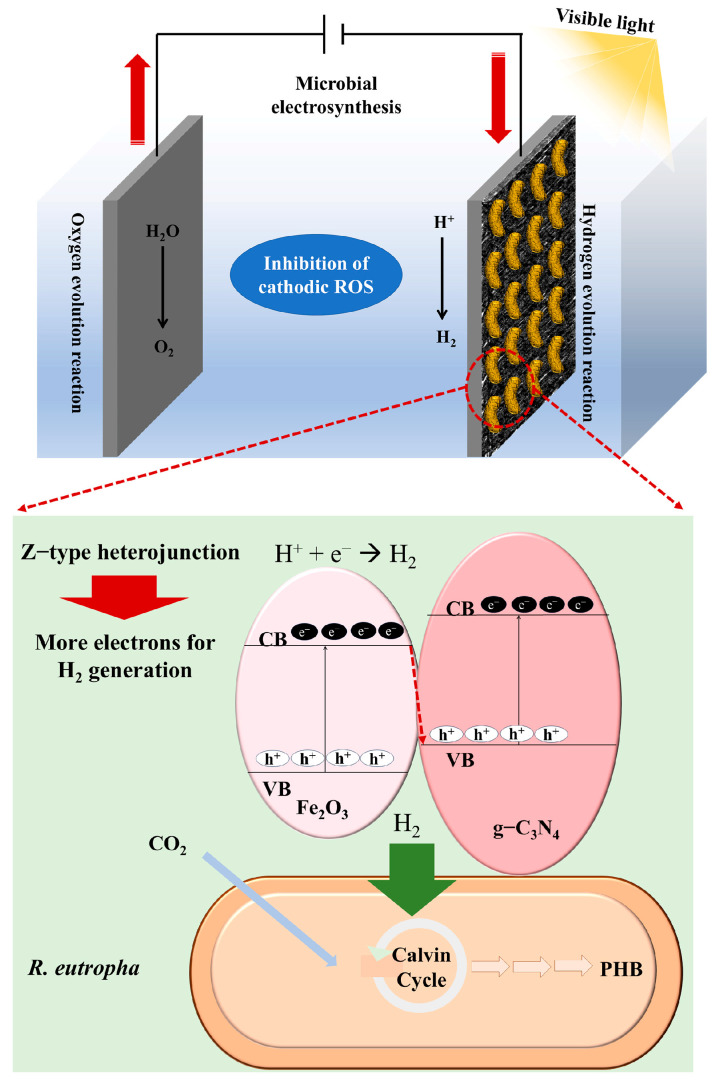
Schematic illustration of the photoelectrochemical (PEC) system coupling a Z-scheme Fe_2_O_3_/g-C_3_N_4_ heterojunction with *C. necator* H16. Under simulated sunlight and external DC power, photogenerated electrons follow a Z-scheme transfer pathway (red arrows) from the conduction band of Fe_2_O_3_ to the valence band of g-C_3_N_4_. This facilitates efficient charge separation to reduce protons into H_2_ (green arrows), which subsequently drives the bacterial Calvin Cycle to fix CO_2_ (blue arrow) into PHB.

To overcome the limitations of single-component materials, researchers have developed composite photosensitizers [107] and explored electrochemically assisted hybrid systems, aiming to enhance electron transfer efficiency and system stability by constructing heterojunctions or multi-component interfaces [108]. Composite photosensitizers optimize performance by coupling different materials to leverage synergistic effects. For instance, a CoP-Fe_2_O_3_/g-C_3_N_4_ composite material forms a Z-scheme heterojunction (Figure 6). This structure not only broadens the absorption range of visible light but also effectively promotes the separation and migration of photogenerated electron-hole pairs, thereby improving photocatalytic hydrogen production efficiency.

Within an electrochemically assisted bio-hybrid system (applying a potential of −0.9 V vs. RHE), this material served as the cathode, supplying reducing power for the autotrophic synthesis of PHB by *C. necator* H16, achieving a PHB productivity of 0.142 g/L [92]. Beyond direct photocatalytic systems, electrochemical-biohybrid systems offer an alternative pathway.

In these systems, cathode materials catalyze the production of reduced substances like H_2_ or formate under an applied potential. Microorganisms then utilize these compounds as energy and reducing power for CO_2_ fixation and PHB synthesis. While not functioning as direct photosensitizers, these materials share the core function of catalyzing and transferring electrons.

For example, a Ni-decorated carbon nanotubes cathode achieved a PHB titer of 0.477 g/L at −1.2 V (Table 6). A Cu/NiMo cathode reached 0.487 g/L PHB at a lower overpotential of −0.69 V (Table 6). These results indicate that rational material composition and interface engineering can synergistically enhance interfacial electron transfer efficiency between carbon-based materials and metal components, providing microorganisms with a more sustainable supply of reducing power.

A comparison of material properties reveals that there is currently no ideal photosensitive material capable of simultaneously meeting the requirements of high efficiency, high biocompatibility, and high stability. Although inorganic semiconductors exhibit high electron yield, they possess significant biotoxicity [113]; organic semiconductors offer good biocompatibility but limited electron yield; composite materials can balance these properties, yet their preparation is complex. This performance trade-off underscores the necessity for further development of novel photosensitive materials, particularly the need to create a new generation of photosensitive nanomaterials characterized by small size, high biocompatibility, and the ability to enter cells and directly participate in metabolic processes.

It is noteworthy that the selection of photosensitive materials must also consider their compatibility with microbial metabolic networks. In the *C. necator* H16 system, photogenerated electrons need to effectively drive the NADPH regeneration process, which requires the material’s reduction potential to match that of the NADP^+^/NADPH redox couple (−0.32 V vs. SHE). Concurrently, critical factors for material design include the dispersion stability of the material in the fermentation environment, its interactions with cell membrane structures, and its impact on other metabolic pathways.

### 3.4. Photoelectric Conversion and Quantum Efficiency Analysis

Regarding the conversion of standardized monochromatic photons into photo-generated electrons and NADPH, existing literature has not yet established a specific correlational analysis between photo-generated electrons and NADPH. In the fields of biosynthesis and microbial fuel cells (MFCs), the prevailing methodology for quantifying the relationship between photons generated by photosensitizers and the resulting electrons is through External Quantum Efficiency (EQE), also referred to as photoelectric conversion efficiency [114].

#### 3.4.1. Incident Photon-to-Current Conversion Efficiency (IPCE)

External Quantum Efficiency, commonly designated as EQE or IPCE, represents the ratio of collectable electrons or emitted photons to the total number of incident photons on the surface of the photosensitizer. This metric accounts for optical losses, such as reflection and transmission, as well as electrical losses, including recombination resistance. The standard measurement procedure involves scanning wavelengths using a monochromator and calibrating light intensity with a standard detector to record photocurrent or optical power. Factors contributing to an IPCE of less than 100% primarily include surface reflection, interface recombination, series resistance, and spectral mismatch [115].

EQE serves as a critical technique for evaluating the capacity of photosensitizers to convert incident photons into photocurrent across different wavelengths. It reflects the material’s photoelectric conversion efficiency and its wavelength dependence, finding widespread application in domains such as dye-sensitized solar cells, perovskite batteries, and photo-electrocatalytic materials [116]. Furthermore, it is an indispensable tool for characterizing the quantum efficiency of photosensitizers within hybrid systems comprising microbes and photosensitive materials.

The formula for calculating IPCE is given by $IPCE(\%)=\frac{1240\times I}{\lambda \times \phi }\times 100$, where λ is the wavelength of the incident light, I is the photocurrent density at that wavelength, and φ is the power density (intensity) of the incident light.

#### 3.4.2. Optimization Strategies for Hybrid Systems

Research into copper oxide selenium (hybrid CuSe) and graphene oxide composites has demonstrated that hybrid structures can significantly improve solar energy capture efficiency and enhance charge separation capabilities, thereby elevating the overall photoelectric conversion efficiency [117]. Systematic studies indicate that the photoelectric efficiency of such systems exceeds the average of standard thin films by 8% to 10%.

Future material design should focus on improving photoelectric efficiency through several key strategies. Optimization of the band structure and bandgap regulation is essential, as achieving ideal band matching can effectively reduce exciton recombination and improve separation efficiency. Materials engineered to form multi-level Z-scheme heterojunctions have demonstrated significantly superior electron separation effects during photocatalytic reactions [118,119]. Interface modification is another critical area, where interface properties including wettability, affinity, and carrier injection characteristics play a decisive role in determining efficiency. Interface performance can be optimized by constructing electron transport layers and multifunctional intermediate layers [120]. Finally, the enhancement of chemical stability and reproducibility is indispensable for maximizing photoelectric conversion efficiency, with studies on perovskite-type photosensitizers highlighting the need for long-term stability, specifically resistance to water vapor and oxidation [121].

**Figure 7:**
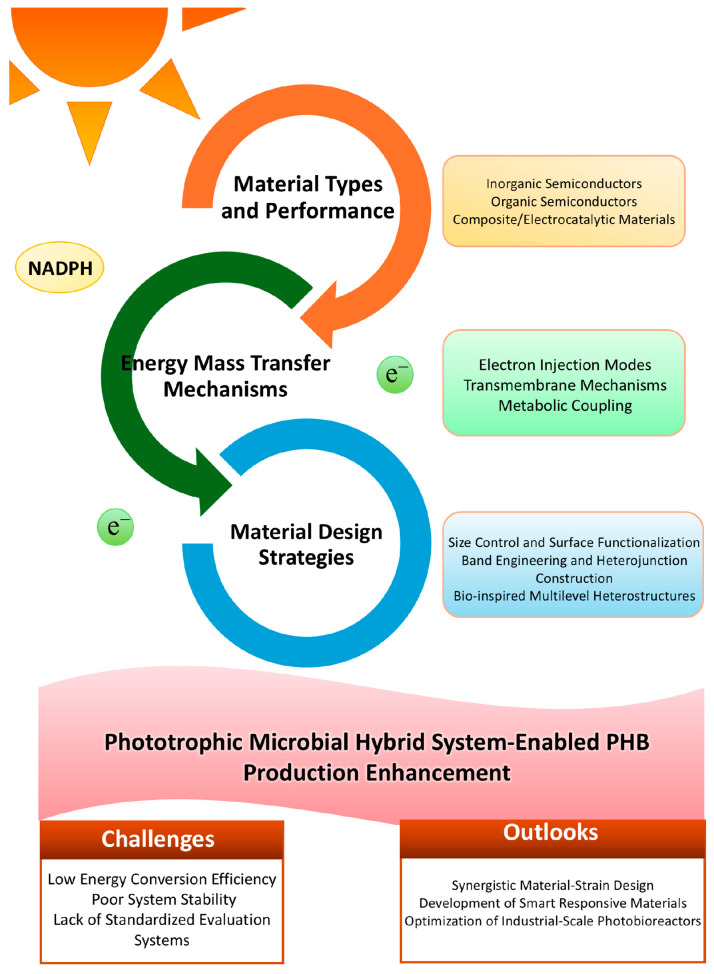
Scope and structure of this review. Different colors represent distinct sections of this review: orange for material types, green for energy transfer mechanisms, blue for material design strategies, and red for PHB production enhancement applications.

In future investigations, advanced quantum dots and nanocomposites should be utilized to deepen the analysis of interface defect capture-passivation mechanisms. This approach aims to further reduce interface carrier losses and promote the enhancement of quantization efficiency in H16 and material hybrid systems [122].The comprehensive scope, design strategies, and future outlooks of this field are illustrated in Figure 7.

## 4. Analysis of Energy and Mass Transfer Pathways at the Material Microbe Interface

Efficient energy and mass transfer between the photosensitive material and the microbe is crucial for achieving high performance in the hybrid system. This process involves multiple steps, including the generation of photogenerated electrons, their transmembrane transport, the regeneration of intracellular reducing power, and ultimately driving the anabolism of PHB. The efficiency of this transfer directly determines the overall performance of the hybrid system [123]. The pathways and regulatory mechanisms of energy and mass transfer at the material microbe interface are systematically analyzed below from three key aspects: electron injection modes, transmembrane mechanisms coupled with reducing power regeneration, and integration with central metabolism.

### 4.1. Electron Transfer Mechanisms in Photo-Driven Biohybrid Systems

The transfer pathways of photogenerated electrons from materials to microbes can be divided into two modes: direct electron transfer and indirect electron transfer. The choice between these modes depends on material properties, microbial surface structures, and the interfacial microenvironment.

#### 4.1.1. Direct Electron Transfer and Intracellular Interactions

**Figure 8:**
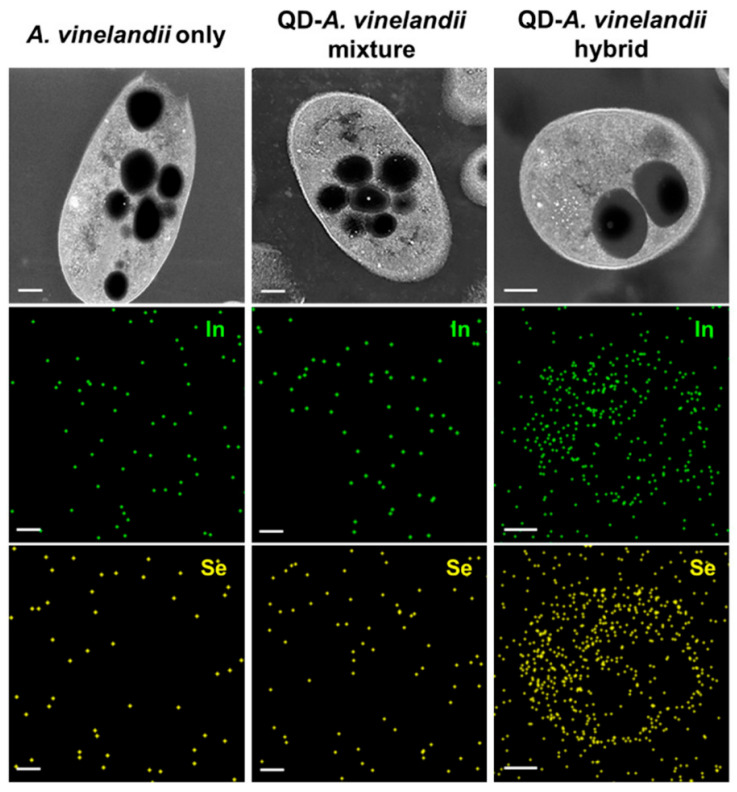
Cross-section HAADF-STEM images and EDS mapping results for Se Kβ and In Lα lines of *A. vinelandii* only, the QD-*A. vinelandii* mixture, and the QD-*A. vinelandii* hybrid cultured in the presence of 200 nM concentration of QDs (scale bar = 200 nm) [124]. Copyright 2025 ACS Publications. All rights reserved.

Direct electron transfer necessitates the formation of a tight physical interface between the photosensitive material and the electron transport chain components located on the outer or inner cell membrane (Figure 8). This physical contact with enzymes such as cytochromes, hydrogenases, or transhydrogenases establishes a conductive interfacial channel for charge injection. For instance, previous investigations demonstrated that a FeCo/NCNT@CF anode with a hierarchical nanostructure significantly promoted direct electron transfer between the electrode and *Geobacter* species which enhanced electron transfer efficiency by over 40%. In photosensitive hybrid systems, size-dependent internalization offers a distinct direct transfer pathway. Materials with sufficiently small dimensions, typically under 10 nm, may traverse the cell membrane via passive diffusion or membrane translocation mechanisms rather than endocytosis. This allows them to access the cytoplasm and achieve intracellular photoelectrocatalysis. A prime example involves the incorporation of ultrasmall gold nanoclusters of approximately 2 nm into the cytoplasm of M. thermoacetica [1]. Super-resolution microscopy and elemental mapping confirmed the intracellular localization of these gold nanoclusters. These internal photosensitizers directly injected electrons into the metabolic network and interacted with enzymes such as transhydrogenases to drive the efficient synthesis of acetic acid from CO_2_.

#### 4.1.2. Indirect Electron Transfer via Redox Mediators

**Figure 9:**
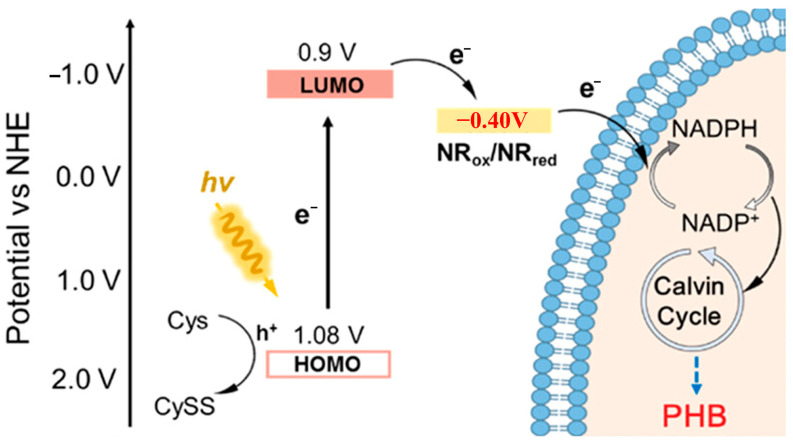
Schematic diagram for Pdots promoting the PHB production of RH16 [2]. The yellow wavy arrow represents light irradiation (hν). Vertical black arrows indicate electron transitions and transfer pathways. The black curved arrows illustrate the redox cycle of coupled with the Calvin Cycle. Copyright 2022 ACS Publications. All rights reserved.

Indirect electron transfer relies on electron shuttles such as riboflavin, quinones, flavoproteins, or synthetic mediators to facilitate electron movement [116]. In this process, photogenerated electrons first reduce these mediators, and the reduced forms subsequently diffuse to the cell surface to deliver electrons to membrane-bound acceptors. While indirect transfer offers wider applicability with less stringent requirements for material-cell spatial arrangement, it incurs greater thermodynamic losses during multi-step transfer. Furthermore, issues regarding mediator stability and biocompatibility often present critical system limitations. Nevertheless, specific hybrid systems have effectively utilized this pathway. For example, in the g-C_3_N_4_/*C. necator* H16 system, Xu et al. suggested that bacterium-secreted riboflavin acts as a shuttle for photogenerated electrons. This mechanism boosted NADPH regeneration and raised PHB production to 6.73 g/L using fructose as a carbon source [95]. Similarly, methyl viologen has been employed as an exogenous shuttle in CdS/*C. necator* H16 systems to transfer electrons from the CdS conduction band to reductases, although applications are constrained by toxicity issues and mediator instability [94]. In the Pdots/RH16 system, NR acts as an electron shuttle, utilizing its redox properties to transfer electrons from outside the membrane to the inside of the bacterial cell [2] (Figure 9).

#### 4.1.3. Experimental Elucidation of Non-H_2_-Mediated Pathways

Under typical autotrophic conditions, *C. necator* H16 primarily conserves energy by oxidizing molecular hydrogen via its Ni-Fe hydrogenases and channeling electrons from H_2_ splitting into the respiratory chain [125]. However, integrated bio-hybrid systems have revealed the potential for alternative non-H_2_-mediated electron uptake pathways. These mechanisms manifest as the direct acceptance of electrons from material surfaces or mediated transfer via redox shuttles located at the cell surface. To rigorously distinguish these pathways from native H_2_ metabolism, current research employs a combinatorial experimental approach. Femtosecond transient absorption spectroscopy has been utilized to capture electron transfer kinetics and revealed that metal sulfides like CdS can achieve direct electronic coupling with microbes through photogenerated surface electron and hole pairs [126]. Additionally, the use of probe molecules such as methyl viologen or p-nitrophenol allows for the monitoring of transfer intermediates where changes in optical absorption quantify electron transfer rates [127]. Crucially, inhibitor experiments employing specific Ni-Fe hydrogenase inhibitors like 2-Bromoethanesulfonate are used to block H_2_ oxidation. Sustained electron transfer and metabolic activity in the presence of such inhibitors provide definitive evidence that the hybrid system operates via an alternative mechanism independent of hydrogen consumption [128,129].

### 4.2. Transmembrane Mechanisms and Reducing Power Regeneration

Electron translocation across the cell membrane constitutes a key rate-limiting step in the energy/mass transfer process, primarily involving pathways mediated by outer membrane porins, catalysis by periplasmic reductases, and mediator-dependent transmembrane transport systems. In the Gram-negative bacterium *C. necator* H16, outer membrane porins such as OprF and porin may provide channels for small-sized nanomaterials or reduced mediators to traverse the outer membrane barrier [130]. After entering the periplasmic space, these electron carriers are subsequently received by periplasmic hydrogenases or cytochrome c redox systems, ultimately completing their transfer into the cytoplasm through the inner membrane electron transport chain. This process has been validated in electrochemical hybrid systems; for example, atomic hydrogen generated by a Ni@CNTs cathode can be directly utilized by *C. necator* H16 via its hydrogenase system, thereby driving CO_2_ reduction and PHB synthesis.

Once electrons enter the cytoplasm, they are typically shuttled by endogenous redox cofactors. NADPH, serving as an essential cofactor for acetoacetyl-CoA reductase in the PHB synthesis pathway, directly determines the efficiency of PHB chain elongation and polymerization. Studies demonstrate that photogenerated electron injection can effectively elevate the intracellular NADPH/NADP^+^ ratio. For instance, in the g-C_3_N_4_/*C. necator* H16 system, the NADPH level increased approximately 1.8-fold under illumination, closely correlating with the 2.12-fold enhancement in PHB production [5]. Furthermore, photoelectrons may potentially enhance the reducing power supply network by activating transhydrogenase to promote the conversion of NADP^+^ to NADPH [131].

### 4.3. Coupling with Central Metabolism

Beyond directly supplying reducing power, photogenerated electrons can indirectly enhance PHB synthesis by modulating the redox state and flux distribution of central carbon metabolism. In *C. necator* H16, carbon fixation and the polymer synthesis pathway form a tightly coupled system. When CO_2_ serves as the carbon source, the organism fixes CO_2_ via the Calvin cycle to generate glyceraldehyde-3-phosphate (GAP). The injection of photoelectrons can accelerate the gluconeogenesis pathway by enhancing the reductive activity of key enzymes such as GAP dehydrogenase, thereby providing more precursor pyruvate for PHB synthesis. Under heterotrophic conditions with a carbon source like fructose, photoelectrons may modulate the NADH/NAD^+^ ratio, activating key enzymes such as phosphoenolpyruvate carboxylase and redirecting carbon flux from energy metabolism towards polymer storage.

The regulation of key metabolic nodes is particularly critical. For instance, the redox states of GAP and pyruvate directly influence the size of the acetyl-CoA pool and the PHB synthesis flux. Photoelectron injection not only promotes the activity of the pyruvate dehydrogenase complex by lowering the intracellular redox potential, thereby enhancing acetyl-CoA generation, but also activates acetyl-CoA carboxylase by elevating NADPH levels, facilitating the synthesis of PHB precursors. Metabolic engineering studies provide evidence for this; for example, overexpressing NAD kinase in *Corynebacterium crenatum* to regulate NADPH levels indeed significantly promoted PHB synthesis [132]. This multi-level regulatory mechanism is fully manifested in the Pdots/*C. necator* H16 system, where photoelectrons not only directly promote NADPH regeneration but also skew carbon flux towards PHB synthesis by regulating the pyruvate node, ultimately achieving the efficient conversion of CO_2_ to PHB [2].

The energy and mass transfer pathways at the material microbe interface represent the core process for enhancing PHB synthesis in photo-driven hybrid systems. Improving its efficiency requires a comprehensive consideration of the synergistic effects among electron injection modes, transmembrane mechanisms, and metabolic coupling. The electron injection efficiency is co-determined by both direct and indirect electron transfer modes. Furthermore, the in situ construction of mineralized photocatalyst interfaces, based on biological templates such as engineered biofilms, offers a novel strategy for optimizing transmembrane electron transfer while ensuring cellular protection. This strategy, together with the regulation of reducing power regeneration, dictates the efficiency of electron integration into the metabolic network. The coupling with central carbon metabolism subsequently amplifies the promoting effect of photoelectrons on PHB synthesis [133]. Through the rational design of the material microbe interface structure and the optimization of the match between electron transfer pathways and metabolic flux, breakthrough progress in energy and mass transfer efficiency can be anticipated, thereby advancing photo-driven biomanufacturing technologies towards practical application.

## 5. Design Strategies for Efficient Photosensitive Materials

To achieve the highly efficient and long-term application of photosensitive material microbe hybrid systems in PHB synthesis, it is essential to overcome the inherent trade-offs among material photocatalytic activity, electron transfer efficiency, and biocompatibility. This necessitates synergistic and rational design across multiple dimensions, including material dimensions, interface properties, energy band structure, and system integration. The core objective is to construct a highly efficient, low-loss electron transfer chain spanning from light harvesting to intracellular PHB synthesis.

### 5.1. Size Control and Surface Functionalization: Towards Intracellular Electron Transfer

Reducing material size to the nanoscale, particularly below 20 nm, is a primary strategy for achieving high-efficiency energy and mass transfer. This approach significantly increases the specific surface area, thereby exposing more active sites for catalytic reactions. Furthermore, ultrasmall dimensions can enable a unique process of “intracellular photoelectrocatalysis”, where nanomaterials can be internalized by the cell and traverse the cell membrane via passive diffusion or other translocation mechanisms to directly inject electrons into the intracellular metabolic network. The feasibility of this sophisticated mechanism is powerfully demonstrated by the work of the Zhang team [134], who introduced ultrasmall gold nanoclusters (<2 nm) into non-photosynthetic bacteria, and is further strongly supported by Koh et al., who provided direct evidence that InP/ZnSe QDs are efficiently internalized by *Azotobacter vinelandii* during cultivation, achieving over 80% cellular uptake, which was crucial for direct electron transfer to intracellular nitrogenase and enhanced light-driven ammonia production (Figure 8) [124]. As confirmed by super-resolution microscopy in the former study, these internalized nanoclusters served as intracellular photosensitizers, directly driving the metabolic pathway for solar fuel production from CO_2_. This prominent example underscores the great promise of size-dependent internalization for achieving highly efficient and direct electron delivery.

**Figure 10:**
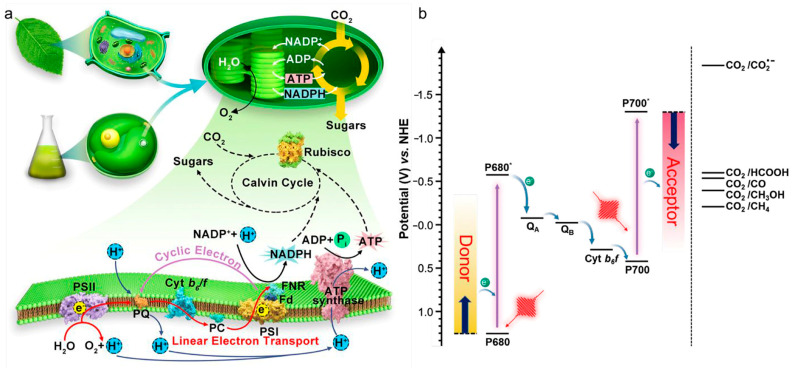
(**a**) Schematic representation of photosynthetic apparatus and the electron transport process. (**b**) Redox potentials of photosynthetic proteins in electron-transfer chain [137]. The asterisk (*) denotes the excited state of the reaction center chlorophylls. Red arrows in (**a**) represent the linear electron transport pathway, while purple arrows indicate cyclic electron transport. Blue curved arrows denote proton (H^+^) movement. Vertical purple arrows in (**b**) represent electron excitation by light, and blue arrows indicate electron transfer steps. Copyright 2021 ACS Publications. All rights reserved.

However, the nanoscale dimension also introduces higher surface energy and potential biotoxicity, necessitating complementary surface functionalization strategies. Recent studies indicate that rationally designing the side-chain chemistry of organic semiconductors (OSCs) can simultaneously achieve excellent interfacial binding and biocompatibility [135]. Zhang et al. developed water-soluble OSCs whose side chains were modified with phosphorylcholine (CP) groups (Figure 10). These groups can form electroneutral CP–PC quadrupole interactions with phosphatidylcholine (PC) [136] in the cell membrane phospholipids, significantly enhancing the interfacial binding affinity between the material and the microbial cell membrane while causing minimal disturbance to the membrane potential, thereby helping to maintain bacterial viability [137]. This surface functionalization strategy, based on specific molecular recognition, not only avoids potential damage to membrane structure associated with traditional electrostatic adsorption but also significantly improves the efficiency of transmembrane electron injection by shortening the electron transfer distance.

### 5.2. Band Engineering and Heterojunction Construction: Optimizing Photogenerated Electron Flux

The intrinsic photoelectronic properties of photosensitive materials determine their capacity for electron generation and output. Band engineering and heterojunction construction serve as core strategies for systematically enhancing this capability. Band engineering, implemented through elemental doping (e.g., introducing P or S into g-C_3_N_4_), defect engineering, or crystal facet control, enables precise modulation of the material’s band structure. This approach not only narrows the bandgap to extend the light absorption range further into the visible spectrum but also adjusts the conduction band position to more negative values than the NAD^+^/NADH reduction potential (−0.32 V vs. SHE), thereby thermodynamically facilitating efficient NADH regeneration. Concurrently, engineered defects can function as electron traps to suppress the recombination of photogenerated electron-hole pairs.

Heterojunction construction involves coupling two or more semiconductor materials with matched band structures. The built-in electric field formed at their interface drives the spatial separation and directional migration of photogenerated electrons and holes, significantly reducing their recombination rate. This strategy, particularly through rational energy level alignment at the heterojunction interface, establishes efficient channels for electron flow, substantially enhancing the quantum efficiency of electron injection into the biological system.

**Figure 11:**
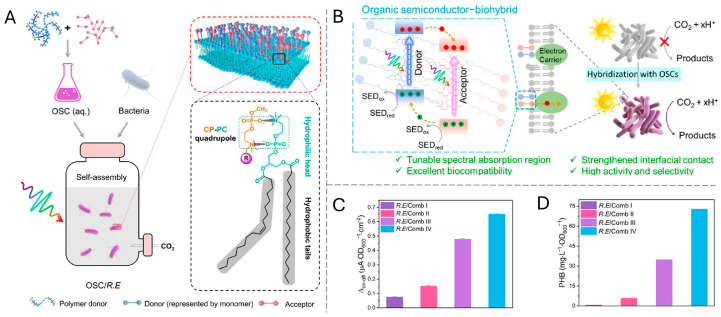
Construction of OSC-bacteria hybrid systems and the effect of ammonium/choline phosphate on their electrical properties.(**A**) Schematic illustration of the construction of OSC/R.E hybrid systems, represented by CP-decorated OSCs, with CP-PC quadrupolar interactions on the cell membrane [137]. (**B**) Optoelectronic properties of OSCs and performance of OSC-biohybrid systems [137]. (**C**) Photocurrent densities of R.E/Comb I-IV under simulated sunlight (AM 1.5G, 100 mW/cm^2^) [137]. (**D**) Corresponding PHB production yields after cultivation under optimized photosynthetic conditions (100 μmol m^−2^ s^−1^, 30 °C, 5 days) [137]. In panels (**C**, **D**), the different colored bars represent specific combinations (e.g., R.E/Comb I–IV) as detailed in the internal legends. Copyright 2025 ACS Publications. All rights reserved.

In the field of organic semiconductors, the construction of donor-acceptor heterojunctions has been demonstrated as an effective approach to enhance charge separation efficiency. For example, Zhang et al. fabricated an efficient bulk heterojunction by combining the broad-spectrum-absorbing donor polymer PBDTTT-C-P with the acceptor small molecule PDI-C-P [138] (Figure 11). This structure not only extended the light absorption range across the entire visible spectrum (380–730 nm) but also, owing to its optimized energy level alignment, promoted the efficient separation and transfer of photogenerated electrons. This resulted in a hybrid system photocurrent density of 0.65 μA OD_600_^−1^ cm^−2^ (Figure 11C), providing a substantial electron flux for efficient CO_2_ photoreduction [137].

### 5.3. Bio-Inspired Multilevel Heterostructure Design: Constructing Electron Transfer Highways

Bio-inspired multilevel heterostructure design, inspired by natural photosynthetic systems, aims to construct efficient micro-devices that integrate light harvesting, electron transfer, and bio-catalysis [139]. Its core principle involves mimicking the synergistic mechanisms of photosystems in photosynthetic membranes: complementary photosensitizers are assembled to form a “light-harvesting antenna,” which then facilitates the directional migration of electrons along an energy level gradient. This electron flow is ultimately coupled efficiently with the microbial metabolic network through precise interface engineering.

**Figure 12:**
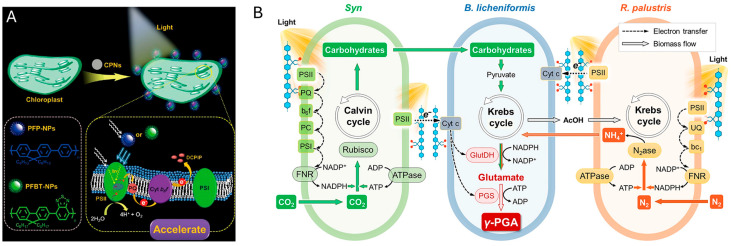
(**A**) Schematic representation of conjugated polymer nanoparticle enhanced light-dependent reaction of chloroplasts [141]. Copyright 2021 ACS Publications. All rights reserved. (**B**) Schematic diagram of electron and substance transfer path in coculture system. PSII, photosystem II; PSI, photosystem I; PQ, plastoquinone; b6f, cytochrome b6f; PC, plastocyanin; FNR, ferredoxin-NADP^+^ reductase; bc1, cytochrome bc1; UQ, ubiquinone; Rubisco, ribulose bisphosphate carboxylase oxygenase; Cyt c, cytochrome c; N_2_ase, nitrogenase; GlutDH, glutamate dehydrogenase; PGS, polyglutamate synthetase [142]. In panel B, green and orange arrows represent carbon and nitrogen metabolic fluxes, respectively. Copyright © 2023 American Association for the Advancement of Science. All rights reserved.

Organic conjugated polymers (CPs) serve as an ideal material platform for this concept, functioning as “artificial antennas” [140] that utilize the Förster resonance energy transfer (FRET) mechanism to funnel underutilized ultraviolet/green light into effective light energy usable by photosynthetic systems, thereby broadening the spectral utilization range [141] (Figure 12A). This strategy has further evolved from single-cell engineering to the construction of multilevel microbial consortia. For instance, Cong et al. designed a solar-powered multi-organism symbiont mimic system using a cationic conjugated polymer (PFP) as a molecular bridge. In this design, PFP not only enhances the photosensitization of Synechocystis but also facilitates direct interspecific electron transfer (DISET) and substance exchange between photoautotrophs (Syn) and heterotrophs (*B. licheniformis* and *R. palustris*). This logic-gated “intercellular electron highway” successfully couples carbon/nitrogen fixation with polypeptide biosynthesis, increasing the production of poly-γ-glutamic acid (γ-PGA) by over 2-fold compared to non-hybrid systems(Figure 12B) [142].

In summary, this bio-inspired strategy, which integrates the functions of artificial antennas and electronic bridges, systematically addresses key challenges such as photogenerated charge separation, migration, and interfacial injection [143]. It marks the transition of the “electron transfer highway” from concept to practice [144], providing a practically viable technological pathway for substantially enhancing solar-to-chemical energy conversion efficiency.

## 6. Conclusions and Outlook

### 6.1. Conclusions

Photosensitive material microbe hybrid systems represent an innovative technological pathway that transcends the scope of traditional fermentation and genetic engineering, effectively addressing the critical bottleneck of insufficient reducing power (NADPH) supply in PHB biosynthesis. Through a systematic review of the research progress in this field, this review draws the following core conclusions. Firstly, photosensitive materials, such as g-C_3_N_4_, CdS, and Pdots, serve as efficient exogenous “light-to-chemical energy” converters. Their core mechanism of action lies in significantly elevating intracellular NADPH levels, primarily via direct or indirect electron transfer modes, thereby effectively driving both the Calvin cycle for carbon fixation and the PHB biosynthetic pathway in production strains like *C. necator* H16. Secondly, the efficiency of energy and mass transfer at the material microbe interface is a key determinant of overall system performance. Specifically, the coupling degree between the transmembrane electron transfer mechanisms and the central carbon metabolic network directly dictates the ultimate conversion efficiency from light energy to PHB.

Furthermore, significant trade-offs currently exist among key performance parameters such as electron yield, biocompatibility, and operational stability across different types of photosensitive materials. No single material yet fulfills all the requirements for practical application. Consequently, future material design must evolve towards rationalization and synergistic integration. This involves employing multidimensional strategies, including precise size control, surface functionalization, band structure optimization, and the construction of biomimetic multi-level heterostructures. Such approaches systematically enhance the material’s light-harvesting capacity, electron separation efficiency, interfacial transfer performance, and biocompatibility, thereby constructing a highly efficient electron transfer chain from light absorption to PHB synthesis.

### 6.2. Outlook

While photosensitive material microbe hybrid systems demonstrate considerable application potential, their transition from laboratory research to industrial application still faces multiple challenges. Future research should seek breakthroughs in the following key directions.

#### 6.2.1. Enhancement of Energy Conversion Efficiency and System Stability

The overall solar-to-chemical energy conversion efficiency of most current hybrid systems remains below 1%, severely constraining their energy economics and industrial prospects. This efficiency bottleneck primarily stems from three aspects, namely the high recombination rate of photogenerated charge carriers in the photosensitive materials themselves, significant electron transfer losses at the material microbe interface, and uneven electron distribution within the microbial metabolic pathways.

Concurrently, insufficient long-term operational stability presents a prominent challenge. Photosensitive materials are susceptible to issues such as photocorrosion, chemical degradation, particle aggregation, or biofouling in the complex fermentation environment, leading to a continuous decline in catalytic activity. Addressing these challenges requires a dual approach. On one hand, efforts must focus on developing novel photosensitive materials with high photostability and self-healing properties, for instance, by constructing core-shell structures or incorporating passivation layers to enhance chemical stability. On the other hand, the comprehensive performance of the system must be improved through innovative photobioreactor design. This includes optimizing the internal illumination system to ensure uniform light distribution, enhancing mass transfer efficiency to guarantee smooth circulation of nutrients and products, and exploring continuous or semi-continuous operation modes to maintain long-term stable system operation.

##### Optimization of Photobioreactor Design

To fully utilize the characteristics of photosensitizers and ensure overall equilibrium from photon transfer to cellular dynamic distribution, the design of modern photosynthetic biohybrid systems must adhere to specific engineering principles [145]. Traditional external light sources are often limited by finite light penetration depths, which leads to insufficient illumination in the central regions of the reactor. Internal illumination systems offer a robust engineering solution to this issue. One approach involves the use of wireless light sources. Embedding Wireless Light Emitters (WLE) within bubble column reactors can achieve a uniform distribution of light intensity [146]. This geometry is particularly effective in dense cellular environments for enhancing bacterial light-driven metabolic efficiency. Another method is optical fiber illumination, which utilizes light transmission technology to introduce light directly into the depths of the reaction medium. This strategy allows precise regulation of directionality and intensity, thereby optimizing the photon supply in high-density microalgae and bacterial cultures [147,148].

The geometric design of the reactor is a critical parameter for light distribution. Flat-plate photobioreactors maximize light intensity per unit surface area through a flattened design, significantly reducing light scattering and attenuation coefficients [149]. Rotating cylinder reactors enhance light distribution uniformity by rotating the reactor wall surface, and they utilize dynamic mixing to minimize cell shading issues [150]. Internal multi-source cylindrical reactors, such as airlift reactors, combine external illumination with multiple internal lamp tubes to achieve a homogenized light field [151]. The optical path length directly dictates the number of photons accessible to cells. Optimization strategies to balance path length with cell density include the following.

Path length reduction is one method; in high-biomass-density environments, utilizing shorter optical paths increases the optical power density per unit volume [152]. Medium modification, such as adding highly transparent media to the liquid, reduces scattering losses and thereby enhances penetration depth [152]. Cell density management is also crucial. High-density cultures often suffer from the “light shading effect,” which reduces photon utilization efficiency [153]. To mitigate this, cell density distribution must be matched with lighting design through mathematical modeling. Fed-batch strategies or layered illumination techniques can dynamically regulate the balance between cell density and photon supply across different reactor zones [154].

Uniform distribution of photon flux is essential for high-efficiency photobiochemical reactions [155]. Engineering metrics indicate that photon flux distribution requires integrating strong light sources (e.g., LED arrays) with light-control algorithms. Simulation approaches, such as the two-flux model, should be employed to determine optimal light distribution patterns that synchronize light absorption with cell proliferation, ensuring that photon input matches the metabolic capacity of the hybrid system.

Future designs must also address reactor photostability under long-term irradiation and investigate micro-scale light field control to manage distribution at the molecular level [156]. Balancing these engineering parameters with economic scalability remains the key challenge for moving from laboratory prototypes to industrial application. Combining the characteristics of diverse photosensitizers with customized reactor designs will provide more sustainable solutions for efficient microbial production, meeting the demands of future industrial applications [157].

#### 6.2.2. Establishment and Refinement of Standardized Evaluation Systems

This interdisciplinary research field currently suffers from a critical lack of universally adopted protocols for the standardized testing and evaluation of photosensitive material performance and hybrid system outputs. This deficiency makes effective cross-comparison of data from different research teams and objective assessment of technological merits exceedingly difficult. Specifically, unified testing standards have yet to be established for key material performance parameters, including the quantum efficiency for electron injection and biocompatibility, where the latter requires a clear distinction between impacts on microbial growth and metabolic activity. Concurrently, consistent calculation benchmarks are absent for critical system output metrics, including the PHB yield based on actual incident light energy and carbon conversion efficiency. Therefore, creating a multi-level, standardized performance evaluation framework that encompasses material properties, interfacial processes, biological metabolism, and overall system performance is paramount. Such a framework will provide a clear benchmarking standard for researchers in the field, significantly accelerating the screening and development of high-performance photosensitive materials and optimized process strategies.

#### 6.2.3. Synergistic Design and Dynamic Regulation Strategies for Materials and Strains

Future research paradigms must transition from simple physical mixing of material and strain to a collaborative rational design of “material–strain” systems. This requires deep integration of materials science and synthetic biology. Specifically, advanced synthetic biology tools can be employed to precisely engineer production strains. This includes enhancing their outer membrane electron uptake systems through methods such as overexpressing specific porins or cytochromes, rewiring central carbon metabolic networks to more efficiently channel carbon flux toward PHB synthesis, and even designing entirely novel electron utilization pathways to genetically optimize the compatibility between the strain and the photoelectron characteristics of the material. Conversely, efforts should focus on developing smart materials capable of responding to biological environmental signals such as external light illumination, pH, or redox potential. This enables dynamic, spatiotemporal regulation of the PHB synthesis pathway. For instance, designing a smart material that controllably releases electron mediators under specific light wavelengths would allow for the on-demand and precise initiation and termination of the NADPH regeneration process.

#### 6.2.4. System Adaptation and Engineering Optimization for Industrial Scenarios

Ultimately, the successful industrialization of hybrid systems hinges on their technical feasibility and economic competitiveness within real industrial environments. Future system designs must fully address the specific demands of industrial applications by prioritizing the use of industrial waste gases, such as CO_2_ emissions from coal-fired power plants or steel mills, and various wastewaters, including organics-rich streams from agricultural or food processing effluents, as low-cost carbon sources to substantially reduce production costs. The process must be engineered to maintain system stability under non-axenic or semi-continuous operation modes, thereby significantly cutting sterilization energy demands and operational complexity. At the scale-up level, key engineering challenges require systematic solutions, particularly overcoming light transfer limitations including penetration depth and distribution uniformity in large-scale photobioreactors, improving mass mixing efficiency for high cell-density cultures, and enabling low-cost extraction and purification of the downstream PHB product. Only through the deep integration and synergistic innovation of materials science, microbiology, and process engineering can photo-driven biomanufacturing truly bridge the gap from laboratory proof-of-concept to practical industrial application, offering a viable technological pathway for the green bioeconomy.

## Figures and Tables

**Table 1:** Microorganisms Related to PHB Production.

Microorganism	PHB Yield	Nutrient Source	Literature Source
*Bacillus megaterium* LSRB 0103	0.93 g/L	Corn starch and urea	[20]
*Halomonas* sp.YLGW01	2.9 g/L	Acetic acid	[21]
*R. eutropha* ATCC17697	36 g/L	CO_2_ (anaerobic fermentation)	[22]
*Serratia nematodiphila* MB307	4.8 g/L	Glucose	[23]
*Acidovolax* sp. A1169 (psychrophile, 15 °C)	2 g/L	Fructose or mannitol	[24]
*C*. *necator* H16	15.4 g/L	Rubber seed oil as the carbon source and urea as the nitrogen source	[25]
*R. eutropha* H16	12.2 g/L	Sugars from carob pods	[26]
*R. eutropha* H16	4.5 g/L	Corn stover	[27]
*C*. *necator* H16	0.69 g/L	CO_2_ produced during the ethanol fermentation process	[28]

**Table 2:** Comparison of Core Characteristics of Major PHB Synthesis Methods.

Method	Principle	Advantages	Disadvantages	Reference
Chemical Synthesis	Ring-opening polymerization of chemical monomers like β-butyrolactone	Precise control over polymer structure and molecular weight	Dependent on petroleum-based feedstocks; poor sustainability	[32]
Transgenic Plant	Integration of PHB biosynthetic pathways into plants to utilize photosynthesis	Potential for low-cost production via direct CO_2_ fixation	Low polymer yield, high extraction costs, impaired plant growth	[33]
Chemo-enzymatic Method	Combination of chemical hydrogenation and enzymatic conversion	High atom economy, reduces carbon emissions	Complex multi-enzyme coordination, challenges with catalyst cost and stability	[15]
Microbial Fermentation	Utilization of microbial cells as natural bioreactors for metabolic conversion	Mature technology, mild conditions, excellent biocompatibility and biodegradability	High production costs, relatively low product yield	[34]

**Table 3:** Performance Characteristics and Cost Comparison of Four Bioplastics.

Material	Biodegradability	Marine Biodegradability	Biocompatibility	Processability	Production Cost
PLA	Requires specific conditions	Difficult to degrade	Limited for medical applications	Easy to process	Moderate (~$2200/ton)
PBAT	Good biodegradability	Long degradation cycle	Generally good	Good film-forming ability	Relatively low (~$1800/ton)
PBS	Good biodegradability	Long degradation cycle	Generally good	Good heat resistance	Moderate (~$2100/ton)
PHB	Completely biodegradable	Completely biodegradable	In vivo degradable	Poor thermal stability	High (~$21,800/ton)

**Table 4:** Summary of Main Advantages and Disadvantages of Four Bioplastics.

Material	Key Advantages	Key Disadvantages
PLA (Polylactic Acid)	High rigidity and tensile strength Good transparency and processability High biosafety	Poor toughness and brittleness Requires specific industrial composting conditions for degradation
PBAT (Polybutylene Adipate Terephthalate)	Excellent flexibility and elongation at break Good film-forming ability Completely biodegradable	Low gas barrier properties Relatively low mechanical strength
PBS (Polybutylene Succinate)	Good heat resistance Excellent biocompatibility Strong thermal stability	Relatively low hydrolysis resistance Moderate mechanical properties
PHB (Polyhydroxybutyrate)	Completely biodegradable in various environments Excellent biocompatibility Biodegradable in marine environments	High production cost Inherent brittleness and narrow processing window

**Table 6:** Autotrophic PHB Production by C. necator H16 via Electrochemical Hydrogen Evolution.

Cathodic Hydrogen Evolution Material	PHB Yield	Cathodic Potential	Literature Sources
PTh-NSi	0.663 g/L	−1.2 V	[109]
Ni@CNTs	0.477 g/L	−1.2 V	[93]
Cu/NiMo	0.487 g/L	−0.69 V	[110]
Ni@N-C	0.386 g/L	−0.74 V	[111]
Carbon cloth (assisted by formate dehydrogenase, FDH)	0.485 g/L	−0.6 V	[112]
CoP-Fe_2_O_3_/g-C_3_N_4_	0.142 g/L	−0.9 V	[93]

## Data Availability

No new data were created or analyzed in this study. Data sharing is not applicable to this article.
